# A Novel Wireless Power Transfer-Based Weighed Clustering Cooperative Spectrum Sensing Method for Cognitive Sensor Networks

**DOI:** 10.3390/s151127760

**Published:** 2015-10-30

**Authors:** Xin Liu

**Affiliations:** College of Astronautics, Nanjing University of Aeronautics and Astronautics, Nanjing 210016, China; E-Mail: liuxinstar1984@nuaa.edu.cn; Tel.: +86-1507-7874-566

**Keywords:** cognitive sensor network, cooperative spectrum sensing, wireless power transfer, spectrum access, resource optimization

## Abstract

In a cognitive sensor network (CSN), the wastage of sensing time and energy is a challenge to cooperative spectrum sensing, when the number of cooperative cognitive nodes (CNs) becomes very large. In this paper, a novel wireless power transfer (WPT)-based weighed clustering cooperative spectrum sensing model is proposed, which divides all the CNs into several clusters, and then selects the most favorable CNs as the cluster heads and allows the common CNs to transfer the received radio frequency (RF) energy of the primary node (PN) to the cluster heads, in order to supply the electrical energy needed for sensing and cooperation. A joint resource optimization is formulated to maximize the spectrum access probability of the CSN, through jointly allocating sensing time and clustering number. According to the resource optimization results, a clustering algorithm is proposed. The simulation results have shown that compared to the traditional model, the cluster heads of the proposed model can achieve more transmission power and there exists optimal sensing time and clustering number to maximize the spectrum access probability.

## 1. Introduction

Based on cognitive radio (CR), cognitive sensor networks (CSN) have been recently proposed as a way to overcome the shortage of wireless spectrum resources depending on two important functionalities: Spectrum sensing and adaptation [1]. In a CSN, the cognitive node (CN) firstly senses the spectrum environment for learning the occupation status of the frequency spectrum allocated to the primary node (PN) in a primary network. Once an idle spectrum is found, to improve the spectrum access and decrease the interference to the PN, the CN may adapt its transmission parameters for operating in the new spectrum [2,3].

In CSN, spectrum sensing is very important. A low detection probability will make the CN cause interference to the PN, while a high false alarm probability will cause the CN to lose an opportunity for using the idle spectrum [4]. Since the location, structure and strength of the PN signal are often unknown to the CN, energy detection serves as the optimal single-node spectrum sensing method without acquiring any information of the received signal [5]. However, when the PN is in severe fading and shadow conditions, the energy detection performance will degrade greatly, thus cooperative spectrum sensing has been proposed to cope with this problem by allowing multiple CNs to sense the PN collaboratively [6]. In cooperative spectrum sensing, all the CNs sense the PN locally and independently, and then forward their local sensing information to a fusion center that combines all this sensing information to obtain a final decision on the presence of the PN [7]. In [8], a cooperative spectrum sensing based on weight fusion is proposed to improve the sensing performance. In [9], the authors consider the linear combination weights for the fusion center that together maximize the global detection probability. However, minimizing the false alarm probability is not considered, which may improve spectrum access of the CSN. In [10], a sensing-throughput tradeoff model is proposed to maximize the throughput of the CSN through selecting an optimal sensing time. However, the cooperative time, namely, cooperative overhead, may reduce the transmission time as the number of cooperative nodes increases [11]. In [12], a cooperative multiband CSN is considered, where the CNs are allowed to use some of the transmission slot to relay PN data through cooperative communication, while using the remnant of the transmission slot to forward its own data over multiple sub-bands in each frame. In [13], the authors examine the energy-throughput tradeoff for cooperative spectrum sensing and formulate an optimization problem for the tradeoff between energy and throughput of CNs based on spectrum sensing efficiency. However when collaborative CNs are above a certain number, increasing the number of CNs cannot improve the detection performance significantly, and instead, will cause more energy consumption and delay. Hence in [14,15], to improve sensing performance and decrease cooperative overhead and energy consumption, a cluster-based cooperative spectrum sensing is proposed, where the CNs are divided into several clusters and the most favorable nodes are selected as the cluster heads for performing cooperative spectrum sensing. However compared to the common CNs, the cluster heads will consume more energy for cooperative spectrum sensing, yielding to decrease its transmission power. In [16], the authors focus on the performance analysis and comparison of hard decision and soft decision based approaches for cooperative spectrum sensing in the presence of reporting channel errors. In [17,18], the authors have denoted that several imperfections such as noise uncertainty, channel/interference uncertainty, transceiver hardware imperfections, signal uncertainty, synchronization issues, *etc.*, may severely deteriorate spectrum sensing performance. However, the clustering cooperative spectrum sensing can effectively solve some of the problems such as channel imperfections, signal/noise uncertainty, *etc*.

Wireless power transfer (WPT), which enables the receivers to transfer energy from propagating electromagnetic waves in radio frequency (RF), has recently gained attention in both academia and industry [19]. A WPT system allows the energy to flow between two points in space without any interconnecting wires, through installing a RF energy-conversion circuit that converts the collected electromagnetic energy to the electrical energy for supplying the system operations [20]. In [21], the authors consider a stochastic-geometry model in which PNs and CNs are distributed as independent homogeneous Poisson point processes and communicate with their intended receivers at fixed distances; each PN is associated with a guard zone to protect its intended receiver from CN interference, and at the same time delivers RF energy to CNs located in its transferring zone. However, the transferred energy can be used only for CN transmission but not for spectrum sensing. Most of the research work on spectrum sensing focuses on using the received signal energy to sense frequency spectrum, but the energy cannot be utilized to supply the sensing operation, thus yielding both energy consumption and sensing cost. The contributions of the paper can be listed as follows:

(1) The paper firstly combines WPT and spectrum sensing and proposes a novel WPT-based weighed clustering spectrum sensing, in which the common CNs of each cluster receive the RF energy of the PN signal that is then transferred to the cluster head, in order to supply the energy consumption of sensing and cooperation of the cluster head.
(2) In our proposed model, fewer nodes will participate in cooperative spectrum sensing, thus the energy and time used for spectrum sensing may decrease greatly. Moreover, the common CNs may transfer the received wireless power to the cooperative nodes, thus the transmission power of the cooperative nodes can be guaranteed.
(3) A joint resource optimization problem is formulated to maximize the spectrum access probability of the CSN through jointly optimizing sensing time and clustering number. With the solutions of the proposed optimization problem, the CSN can obtain larger spectrum access probability while guaranteeing the spectrum sensing performance.

The rest of this paper is organized as follows: In Section 2, both energy detection and weighed cooperative spectrum sensing are introduced. WPT-based clustering cooperative spectrum sensing and the joint resource optimization problem are presented in Section 3. Following this, the clustering algorithm is described in Section 4. Simulations and discussions are provided in Section 5 and the conclusions are finally drawn in Section 6.

## 2. Spectrum Sensing Models

Common notation as summarized in Table 1 is used throughout this paper.

**Table 1:** Notation.

Symbol	Denotation	Symbol	Denotation
$y_{i}$	received signal by CN*i*	$H_{0}$	absence of PN
$H_{1}$	presence of PN	** s(t)	PN signal
$p_{s}$	power of PN signal	n(t)	Gaussian noise
$\sigma_{n}^{2}$	nosie variance	$h_{i}(t)$	channel gain from PN to CN*i*
*M*	number of signal samples	$f_{s}$	sampling frequency
τ	sensing time	$\gamma_{i}$	sensing signal to noise ratio
$\lambda_{i}$	sensing threshold	Ω(y)	energy statistic
$\omega_{i}$	combined weight	$P_{i}^{f}$	single false alarm probability
$P_{i}^{d}$	single detection probability	$Q^{f}$	cooperative false alarm probability
** $Q^{d}$	cooperative detection probability	μ	electromagnetism-to-electricity conversion efficiency
$Q^{m}$	cooperative missed detection probability	$P^{e}$	BER of the reported sensing information
*D*	number of CNs	*K*	number of cluster heads
$P_{Acc}$	spectrum access probability	$E_{i}^{Head}$	transferred energy of cluster head
$E_{i}^{Comm}$	transferred energy of common CN	T	frame length
τ	sensing time	ε	average cooperative time overhead
η	electricity-to-electromagnetism conversion efficiency	$p_{t}$	information transmission power

### 2.1. Energy Detection

In CSN, each CN finds it difficult to obtain any prior information of the PN signal, thus energy detection is used to sense the PN without needing any information about the detected signal. By comparing the energy statistic of the PN signal to a properly set decision threshold, energy detection declares the presence of the PN when the energy statistic is above the threshold, while deciding the absence of the PN when the energy statistic is below the threshold. Owing to its operating principle that measures the signal energy, the energy detection performance is independent on any prior information of the detected signal. The detected signal $y_{i}$ received by the CN *i* is given by a binary hypothesis problem as follows [22]:

(1) $$y_{i}(t)=\left\{ \begin{array}{l} n(t),\;H_{0}\\ h_{i}(t)s(t)+n(t),\;H_{1} \end{array}\right.\;,\;t=1,2,...,M$$

where *M* is given by *M = τfs*. From Equation (1), the energy statistic of $y_{i}$ is given as follows:

(2) $$\text{Ω}(y_{i})=\frac{1}{M}\sum_{t=1}^{M}{\left\|y_{i}(t)\right\|}^{2}$$

Since *y*(1), *y*(2), …, *y*(*M*) are independently and identically distributed, Ω(y) obeys the Gaussian distribution with a large *M* as follows:

(3) $$\text{Ω}(y_{i})\sim \left\{ \begin{array}{l} N\left(\sigma_{n}^{2},\;\;\sigma_{n}^{4}/M\right),\;H_{0}\\ N\left(\left(1+\gamma_{i}\right)\sigma_{n}^{2},\;\;{\left(1+\gamma_{i}\right)}^{2}\sigma_{n}^{4}/M\;\right),\;H_{1} \end{array}\right.$$

where sensing SNR $\gamma_{i}=h_{i}^{2}p_{s}/\sigma_{n}^{2}$. Comparing $\text{Ω}(y_{i})$ to a threshold $\lambda_{i}$, the probabilities of false alarm and detection are given as follows:

(4) $$\left\{ \begin{array}{l} P_{i}^{f}=P_{r}\left(\text{Ω}(y_{i})\geq \lambda_{i}|H_{0}\right)\;=Q\left(\left(\frac{\lambda_{i}}{\sigma_{n}^{2}}-1\right)\sqrt{\tau f_{s}}\right)\\ P_{i}^{d}=P_{r}\left(\text{Ω}(y_{i})\geq \lambda_{i}|H_{1}\right)=Q\left(\left(\frac{\lambda_{i}}{\sigma_{n}^{2}(\gamma_{i}+1)}-1\right)\sqrt{\tau f_{s}}\right) \end{array}\right.$$

where the function $Q(x)=\frac{1}{\sqrt{2\pi }}\int_{x}^{+\infty }\exp\left(-z^{2}/2\right)\text{ d}z$.

### 2.2. Weighed Cooperative Spectrum Sensing

If the PN is experiencing severe fading and shadowing conditions, the energy detection performance can be greatly decreased. One of the ways to improve spectrum sensing reliability is through cooperative spectrum sensing. Cooperative spectrum sensing is done by combining all the energy statistics from the local sensing of the CNs and performing energy detection to make a final decision at the fusion center. The performance of cooperative spectrum sensing is improved by achieving a sensing diversity gain that is provided by the different sensing paths from multiple CNs. Even though one node has failed to detect the presence of PN, the other nodes may also help to detect the PN.

In centralized cooperative spectrum sensing, a fusion center combines the collected local sensing information of all the cooperative CNs and decides which channel should be used. Then the decision is broadcast to all the CNs. The centralized cooperative spectrum sensing can control the cooperative CNs effectively, collect enough spectrum sensing information and thus greatly improve the sensing performance. In distributed cooperative spectrum sensing, the CNs exchange their sensing information mutually and each CN only maximizes its own spectrum sensing performance, thus both information share degree and spectrum sensing performance are very low [23]. Moreover, without the management of a fusion center, the sensing information will be exchanged frequently among the CNs and thus the sensing time may be very long. Hence, in this paper we adopt centralized cooperative spectrum sensing to obtain more sharing information.

**Figure 1:**
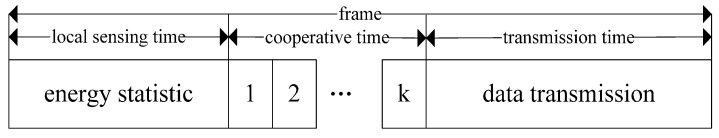
Frame structure of CSN.

The frame structure of cooperative spectrum sensing-based CSN is divided into local sensing time, cooperative time and transmission time, as shown in Figure 1 [24]. In the local sensing time, each CN senses to obtain the local energy statistic of the PN, and in the corresponding time slot of the cooperative time, the CN forwards its energy statistic to the fusion center through a public control channel. An aggregate energy statistic is obtained by combining all the local energy statistics at the fusion center, which is then compared to a set threshold to get the final decision on the presence of the PN. If the PN is determined to be absent, the CSN will transmit data in the transmission time.

**Figure 2:**
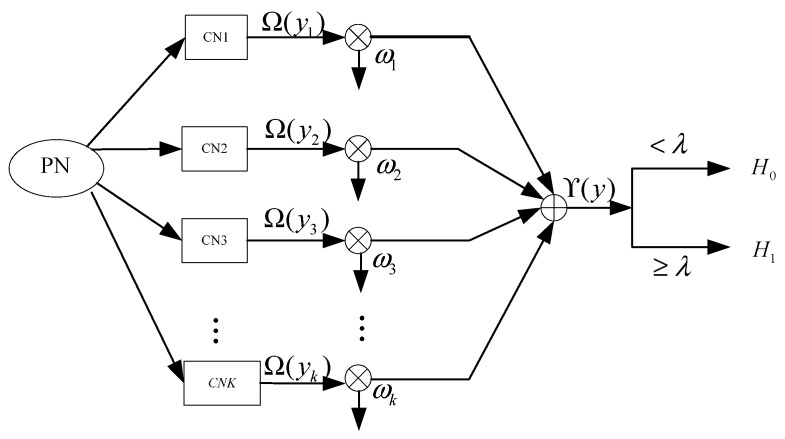
Weighed cooperative spectrum sensing.

The weighed cooperative spectrum sensing is shown in Figure 2. Supposing that there are *k* CNs in the cooperative spectrum sensing, from Equation (2), the combined energy statistic is given as follows:

(5) $$ϒ(y)=\sum_{i=1}^{k}\omega_{i}\text{Ω}(y_{i})$$

where $\omega_{i}$ satisfies $\sum_{i=1}^{k}\omega_{i}^{2}=1$.

If the reporting channel to the fusion center is perfect, substituting Equation (5) into Equation (4), the cooperative probabilities of false alarm and detection are given as follows:

(6) $$\left\{ \begin{array}{l} Q^{f}\;=Q\left(\left(\frac{\lambda }{\sigma_{n}^{2}}-\sum_{i=1}^{k}\omega_{i}\right)\sqrt{\tau f_{s}}\right)\\ Q^{d}=Q\left(\left(\frac{\lambda /\sigma_{n}^{2}-\sum_{i=1}^{k}\omega_{i}(\gamma_{i}+1)}{\sqrt{\sum_{i=1}^{k}\omega_{i}^{2}{\left(\gamma_{i}+1\right)}^{2}}}\right)\sqrt{\tau f_{s}}\right) \end{array}\right.$$

The cooperative missed detection probability is given by *Q^m^ = 1− Q^d^*. *Q^d^* is often set fixedly according to the interference sufferance of the PN, and we try to decrease $Q^{f}$ for improving the spectrum access of the CSN. From Equation (6), $Q^{f}$ is related with *Q^d^* as follows:

(7) $$\begin{array}{l} Q^{f}=Q\left(Q^{-1}(Q^{d})\sqrt{\sum_{i=1}^{k}\omega_{i}^{2}{\left(\gamma_{i}+1\right)}^{2}}+\sqrt{\tau f_{s}}\sum_{i=1}^{k}\omega_{i}\gamma_{i}\right)\\ \;\leq Q\left(Q^{-1}(Q^{d})(\gamma_{\min}+1)+\sqrt{\tau f_{s}}\sum_{i=1}^{k}\omega_{i}\gamma_{i}\right) \end{array}$$

where $\gamma_{\min}=\min\left\{\gamma_{1},\gamma_{2},...,\gamma_{k}\right\}$. Since *Q*(*x*) is a monotonously decreasing function, we can maximize $\sum_{i=1}^{k}\omega_{i}\gamma_{i}$ to minimize $Q^{f}$ as follows:

(8) $$\sum_{i=1}^{k}\omega_{i}\gamma_{i}\leq \sqrt{\sum_{i=1}^{k}\omega_{i}^{2}\sum_{i=1}^{k}\gamma_{i}^{2}}=\sqrt{\sum_{i=1}^{k}\gamma_{i}^{2}}$$

where the maximum is achieved when $\omega_{i}=\gamma_{i}/\sqrt{\overset{k}{\underset{i=1}{\sum }}\gamma_{i}^{2}}$ for *i* = 1, 2, …, *k*. Substituting Equation (8) into Equation (7), the minimal false alarm probability is given as follows:

(9) $$Q_{\min}^{f}=Q\left(Q^{-1}(Q^{d})\xi \left({\left\{\gamma_{i}\right\}}_{i=1}^{k}\right)\text{+}\sqrt{\tau f_{s}\sum_{i=1}^{k}\gamma_{i}^{2}}\right)$$

where $\xi \left({\left\{\gamma_{i}\right\}}_{i=1}^{k}\right)=\sqrt{\overset{k}{\underset{i=1}{\sum }}\gamma_{i}^{2}{\left(\gamma_{i}+1\right)}^{2}/\overset{k}{\underset{i=1}{\sum }}\gamma_{i}^{2}}$. However, if the reporting channel to the fusion center is in fading, from Equation (6), the cooperative probabilities of false alarm and detection are given as follows:

(10) $$\left\{ \begin{array}{l} {\tilde{Q}}^{f}=1-\left(1-Q^{f}\right)\left(1-P^{e}\right)-Q^{f}P^{e}\\ {\tilde{Q}}^{d}=1-\left(1-Q^{d}\right)\left(1-P^{e}\right)-Q^{d}P^{e} \end{array}\right.$$

In a Rayleigh fading channel, the probability density function (PDF) of the sensing SNR of CN*i* is given as follows:

(11) $$f(\gamma_{i})=\frac{1}{\bar{\gamma }}\exp\left(-\frac{\gamma_{i}}{\bar{\gamma }}\right)$$

where $\bar{\gamma }$ is the average SNR of all the CNs. Thus the average cooperative detection probability is given as follows:

(12) $$\begin{array}{l} Q^{d}=\int_{0}^{+\infty }Q^{d}(\gamma_{i})f(\gamma_{i})\text{d}\gamma_{i}\\ \;=Q^{f}+Q\left(\frac{1}{\sqrt{2M}\bar{\gamma }}-Q^{-1}(Q^{f})\right)\exp\left(\frac{1}{2M{\bar{\gamma }}^{2}}-\frac{2}{\sqrt{2M}\bar{\gamma }}Q^{-1}(Q^{f})\right) \end{array}$$

## 3. WPT-Based Clustering Cooperative Spectrum Sensing

### 3.1. Wireless Power Transfer (WPT)

**Figure 3:**
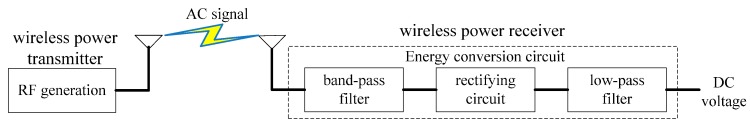
Wireless power transfer model.

WPT generally refers to the transmissions of electrical energy from a power source to one or more electrical loads without any interconnecting wires. WPT can be realized by installing an energy-conversion circuit in the RF front end of a wireless communication system, as shown in Figure 3 [15]. The wireless power receiver acquires the alternating current (AC) signal from the wireless power transmitter and passes the AC signal through a band-pass filter to ensure that it is correctly matched to the rectifying circuit. Then the AC signal is converted to the direct current (DC) signal through a rectifying circuit that involves some number of diodes and capacitors. The DC voltage is finally obtained after filtering out the fundamental and harmonic signals from the DC signal through a low-pass filter. However, some of the signal energy may be reradiated to the outside environment in the energy-conversion process, thus we assume that 0<μ<1 is the electromagnetism-to-electricity conversion efficiency, which is determined by the element characters of the energy-conversion circuit.

### 3.2. Clustering Cooperative Spectrum Sensing

**Figure 4:**
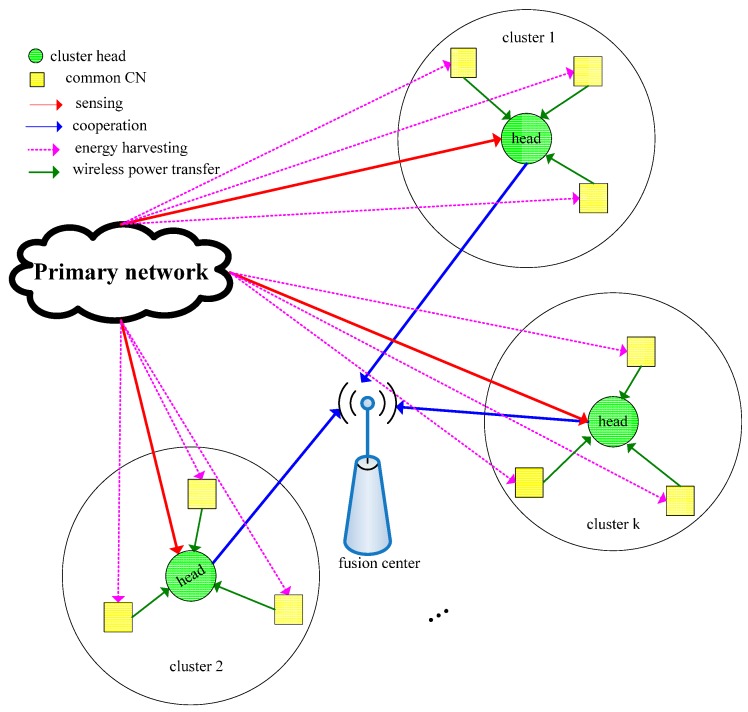
WPT-based clustering cooperative spectrum sensing model.

From Figure 1, it is seen that the cooperative time will be prolonged as the cooperative nodes increases, which yields to shorten the transmission time greatly. In addition, one node, whose path to the fusion center is in fading, may forward false sensing information to the fusion center, thus the cooperative sensing performance is degraded. Hence, to guarantee the transmission quality of the CSN, only some of the CNs with better single sensing performance can be chosen to cooperatively sense the PN. In this paper, WPT-based clustering cooperative spectrum sensing is proposed, in which all the CNs are divided into several clusters, the favorable cluster heads are selected to perform weighed cooperative spectrum sensing and the common CNs of each cluster transfer the RF energy received from the PN signal to the corresponding cluster head, as shown in Figure 4. Since we select the nodes whose mutual distances are nearest as one cluster, the path loss between the common CN and the cluster head is so less that it can be ignored.

With the number of the CNs *D* and the number of the cluster heads (*i.e.*, clustering number) *K*, the number of CNs in each cluster is L=⌈D/K⌉. The CN can access the PN spectrum both in the absence of the PN with accurate detection and in the presence of the PN with missed detection. Hence, the spectrum access probability of the CN is given as follows:

(13) $$P_{Acc}=\frac{T-\tau -K\varepsilon }{T}\left(P(H_{0})\left(1-Q^{f}\right)+P(H_{1})\left(1-Q^{d}\right)\right)$$

where $P(H_{0})$ and $P(H_{1})$ are the probabilities of $H_{0}$ and $H_{1}$, respectively. Since the cluster heads have to perform cooperative spectrum sensing in the sensing time and cooperative time, the cluster heads can only receive the transferred energy of the PN signal in the transmission time, providing that the presence of the PN is detected accurately. Hence, the transferred PN energy of the cluster head is given by:

(14) $$E_{i}^{Head}=\mu P(H_{1})p_{s}h_{i}^{2}Q^{d}\left(T-\tau -K\varepsilon \right),\;i=1,2,...,K$$

When the PN is present, the common CN can receive the transferred energy of the PN signal in the sensing time, cooperative time and transmission time. Hence, the transferred PN energy of the common CN in cluster *i* is given as follows:

(15) $$E_{i}^{Comm}=\mu P(H_{1})p_{s}h_{i}^{2}\left(\tau +K\varepsilon +Q^{d}\left(T-\tau -K\varepsilon \right)\right)$$

Each common CN uses the transferred PN energy to drive the power amplifier to generate the RF signal that is transferred to the cluster head in the cooperative time. Then the cluster head receives the RF energy of *L* − 1 CN signals and converts the RF energy to the electrical energy again. Suppose that the electricity-to-electromagnetism conversion efficiency is 0 < η < 1 that is decided by the element characters of the power amplifier. From Equations (14) and (15), the aggregate transferred energy of the cluster head is given as follows:

(16) $$\begin{array}{l} {\hat{E}}_{i}^{Head}=E_{i}^{Head}+(L-1)\mu \eta E_{i}^{Comm}\\ \;=\mu P(H_{1})p_{s}h_{i}^{2}\left[\mu \eta \left(L-1\right)\left(\tau +K\varepsilon \right)+\left(\mu \eta \left(L-1\right)+1\right)Q^{d}\left(T-\tau -K\varepsilon \right)\right] \end{array}$$

where with $Q^{d}=1$, the maximum transferred energy is given by:

(17) $$\begin{array}{l} {\hat{E}}_{i,\max}^{Head}=\mu P(H_{1})p_{s}h_{i}^{2}\left(\left(\mu \eta \left(L-1\right)+1\right)T-\tau -K\varepsilon \right)\\ \;\leq \mu P(H_{1})p_{s}h_{\max}^{2}\left(\mu \eta \left(L-1\right)+1\right)T \end{array}$$

where $h_{\max}=\max\{h_{1},h_{2},...,h_{D}\}$. With the battery capacity $E_{T}$ and the initial energy $E_{0}$, we have ${\hat{E}}_{i,\max}^{Head}+E_{0}\leq E_{T}$ where we deduce that:

(18) $$T\leq \frac{E_{T}-E_{0}}{\mu P(H_{1})p_{s}h_{\max}^{2}\left(\mu \eta \left(L-1\right)+1\right)}$$

In the traditional clustering cooperative spectrum sensing [12,13], the information transmission power of each cluster head is given as follows:

(19) $$p_{t}=\eta \frac{E_{0}-p_{e}\tau -p_{c}\varepsilon }{T-\tau -K\varepsilon }$$

where $p_{e}$ is the sensing power and $p_{c}$ is the cooperative power. However, in the WPT-based clustering cooperative spectrum sensing, the information transmission power of each cluster head is given as follows:

(20) $${p^{\prime }}_{t}=\eta \frac{{\hat{E}}_{i,\max}^{Head}+E_{0}-p_{e}\tau -p_{c}\varepsilon }{T-\tau -K\varepsilon }$$

where obviously, we have $p_{t}^{\prime }>p_{t}$.

In the proposed model, to guarantee that all the initial energy is used for data transmission of the cluster head, we let ${\hat{E}}_{i,max}^{Head}\geq p_{e}\tau +p_{c}\varepsilon$, thus the transmission power of the cluster head is not less than that of the common CNs. From Equation (17), the expected power level of the transferred energy is given as follows [25]:

(21) $$p_{h}\geq \frac{p_{e}\tau +p_{c}\varepsilon }{\mu P(H_{1})\left(\mu \eta \left(L-1\right)+1\right)T}$$

Thus the distance range of wireless power transfer is given by:

(22) $$\left|d\right|\leq \frac{\lambda }{4\pi }\sqrt{\frac{p_{s}G_{T}G_{R}}{p_{h}L}}$$

where *L* is the path loss factor, *G_T_* is the transmit antenna gain, *G_R_* is the receive antenna gain and λ is the wavelength emitted.

### 3.3. Cooperative Overhead and Wireless Power Transfer Antenna

The cooperative overhead of WPT-based clustering cooperative spectrum sensing happens in the clustering process and sensing process. In the clustering process, the fusion center firstly broadcasts the clustering information to all the cluster heads in the inter-cluster broadcasting slot with the length of $\tau_{1}$, then to avoid mutual interference, the cluster heads broadcast the beacon to their corresponding cluster nodes in *K* special inner-cluster broadcasting slots with the length of $\tau_{2}$, and finally each common CN joins the corresponding cluster according to its received beacon in the grouping slot with the length of $\tau_{3}$. In the sensing process, each common SU transfers its received PN energy to the corresponding cluster head in the energy transfer slot with the length of $\tau_{4}$, then to avoid information conflicts, the cluster heads send their sensing information to the fusion center in *K* special reporting slots with the length of $\tau_{5}$. Thus the total cooperative time overhead is given as follows:

(23) $$\tau_{oh}=\tau_{1}+K\tau_{2}+\tau_{3}+\tau_{4}+K\tau_{5}$$

from which, the average cooperative time overhead of each SU is given by:

(24) $$\varepsilon =\frac{\tau_{oh}}{K}=\tau_{2}+\tau_{5}+\frac{\tau_{1}+\tau_{3}+\tau_{4}}{K}$$

**Figure 5:**
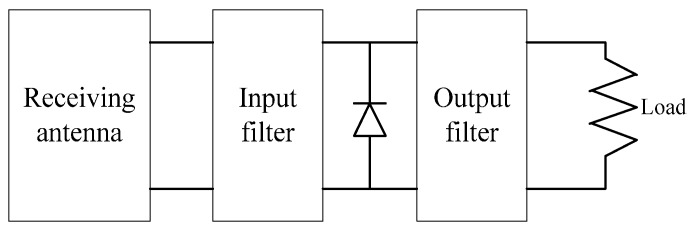
Rectifying antenna structure.

Since both the common CN and cluster head must transfer and receive the RF energy, the WPT antennas adopted by them are often the rectifying antenna that converts RF energy to electrical power, whose structure is shown in Figure 5.

As shown in Figure 1, in the local sensing time, the common CNs transfer RF energy while the cluster heads perform energy detection; in the cooperative time, since each cluster head only uses one of *k* slots to report the sensing information to the fusion center, it can receive the RF energy of the common CNs in the other *k* − 1 slots. Moreover, the cluster head cannot receive the transferred energy in the sensing time because of spectrum sensing, thus it can only receive energy in the transmission slot if the presence of the PN is detected accurately. Accordingly, the cluster head receives the transferred CN energy and PN energy in the cooperative slot and transmission slot, respectively. The transferred energy can be used in the transmission slot for increasing the transmission energy and compensating the sensing consumption, thus the transmission power of the cluster head can be improved.

### 3.4. Joint Resource Optimization

Our goal is to maximize the spectrum access probability of the CSN by jointly optimizing sensing time τ and the clustering number *K*, subject to the constraints that the detection probability is above the lower limit of detection performance and the aggregated transferred energy of the cluster head can supply the electrical energy dissipated in the cooperative spectrum sensing. This optimization problem is given as follows:

(25) $$\begin{array}{l} \underset{\tau ,K}{\max}\;P_{Acc}\left(\tau ,K\right)\\ \text{s.t. }Q^{d}\geq \alpha \\ \;\tau +K\varepsilon \leq T\\ \;1\;\leq K\leq N,\;K\in Z\\ \;{\hat{E}}_{i}^{Head}\geq p_{e}\tau +p_{c}\varepsilon ,\;i=1,2,...,K\\ \;E_{0}\geq p_{e}\tau +p_{\min}(T-\tau ) \end{array}$$

where α is the lower limit of detection probability and $P_{min}$ is the minimum transmission power of the CN. From Equation (7), *Q^f^* and *Q^d^* have the same monotonicity and therefore *P_Acc_* improves as *Q^d^* decreases, *i.e.*, *P_Acc_* achieves the maximum only if *Q^d^ = α*. Substituting *Q^d^ = α* and Equation (9) into Equation (13), *P_Acc_* is rewritten as follows:

(26) $$P_{Acc}=\frac{T-\tau -K\varepsilon }{T}\left(P(H_{0})\left(1-Q\left(Q^{-1}(\alpha )\xi \left({\left\{\gamma_{i}\right\}}_{i=1}^{k}\right)\text{+}\sqrt{\tau f_{s}\sum_{i=1}^{k}\gamma_{i}^{2}}\right)\right)+P(H_{1})\left(1-\alpha \right)\right)$$

Substituting Equations (16) and (26) into Equation (25) and noting that the least transferred energy of the cluster head must satisfy the constraint:

(27) $$\min\left({\left\{{\hat{E}}_{i}^{Head}\right\}}_{i=1}^{K}\right)\geq p_{e}\tau +p_{c}\varepsilon$$

the optimization problem Equation (25) is further deduced as follows:

(28) $$\begin{array}{l} \underset{\tau ,K}{\max\;}P_{Acc}\left(\tau ,K\right)=\frac{T-\tau -K\varepsilon }{T}\left(P(H_{0})\left(1-Q\left(Q^{-1}(\alpha )\xi \left({\left\{\gamma_{i}\right\}}_{i=1}^{K}\right)\text{+}\phi \left({\left\{\gamma_{i}\right\}}_{i=1}^{K}\right)\sqrt{\tau }\right)\right)+\varphi \right)\\ s.t.\;\pi (K)\leq \tau \leq T-K\varepsilon \\ \;E_{0}\geq p_{e}\tau +p_{\min}(T-\tau )\\ \;1\;\leq K\leq N,\;K\in Z \end{array}$$

where $\Phi \left({\left\{\gamma_{i}\right\}}_{i=1}^{K}\right)=\sqrt{f_{s}\overset{K}{\underset{i=1}{\sum }}\gamma_{i}^{2}}$ and φ = P(H_1_)(1 – α). Substituting Equation (16) into Equation (27), π(K) is given as follows:

(29) $$\pi (K)=\frac{\left[p_{c}-\upsilon \left(\theta (1-\alpha )-\alpha \right)K\right]\varepsilon -\alpha \upsilon \left(\theta +1\right)T}{\upsilon \left(\theta (1-\alpha )-\alpha \right)-p_{e}}$$

where $\upsilon =\mu P(H_{1})p_{s}h_{\min}^{2}$, $h_{\min}=\min\{h_{1},h_{2},...,h_{D}\}$ and θ=μη(L−1).

We use the alternating direction optimization (ADO) to solve Equation (28). We formulate two sub-optimization problems about one of the two variables τ and *k* by fixing the other variable with an initial value, respectively, and obtain the optimal solution through optimizing these two sub-optimization problems alternately until both of τ and *k* are convergent [26]. Firstly, we initialize *K* = *K*_0_ where *K*_0_ is any integer within interval (0, min($\frac{T}{\varepsilon }$, *N*)) and select the *K_0_* largest SNRs as ${\left\{\gamma_{i}\right\}}_{i=1}^{K_{0}}$. Then $\xi ({\left\{\gamma_{i}\right\}}_{i=1}^{K_{0}}$ and $\Phi \left({\left\{\gamma_{i}\right\}}_{i=1}^{K_{0}}\right)$ are both constants. Hence, we rewrite Equation (28) as follows:

(30) $$\begin{array}{l} \underset{\tau }{\max\;}P_{Acc}\left(\tau \right)=\frac{T^{\prime }-\tau }{T}\left(P(H_{0})\left(1-Q\left(Q^{-1}(\alpha )\xi \text{+}\phi \sqrt{\tau }\right)\right)+\varphi \right)\\ \text{s.t. }\pi (K_{0})\leq \tau \leq T^{\prime }\\ \;E_{0}\geq p_{e}\tau +p_{\min}(T-\tau ) \end{array}$$

where *T' = T − Kε*. Then we prove that there exists an optimal $\tau_{M}\in \left(0,T^{\prime }\right)$ that yields the maximum P_Acc._ The first-order derivative of *P_Acc_*(τ) is deduced from Equation (30) as follows:

(31) $$\nabla P_{Acc}\left(\tau \right)=-\frac{1}{T}\left(P(H_{0})\left(1-Q\left(Q^{-1}(\alpha )\xi \text{+}\phi \sqrt{\tau }\right)\right)+\varphi \right)+\frac{\left(T^{\prime }-\tau \right)P(H_{0})\phi }{2T\sqrt{2\pi \tau }}\exp\left(-\frac{{\left(Q^{-1}(\alpha )\xi \text{+}\phi \sqrt{\tau }\right)}^{2}}{2}\right)$$

Obviously:

(32) $$\left\{ \begin{array}{l} \underset{\tau \to T^{\prime }}{\lim}\nabla P_{Acc}\left(\tau \right)<-\frac{P(H_{0})}{T}\left(1-Q\left(Q^{-1}(\alpha )\xi \right)\right)<0\\ \underset{\tau \to 0}{\lim}\nabla P_{Acc}\left(\tau \right)=O\left(\frac{1}{\sqrt{\tau }}\right)=+\infty \end{array}\right.$$

where we have used the fact that *Q*(*x*) is a decreasing function and upper bounded by 1. Equation (32) means that *PAcc*(τ) increases when τ is small and decreases when τ approaches *T'*, thus there is a maximum point $\tau_{M}$ within interval (0, *T'*), which can be obtained through using the half searching algorithm. Then from $E_{0}\geq p_{e}\tau +p_{min}\left(T-\tau \right)$, we get $\tau \geq \tau_{l}$ where:

(33) $$\tau_{l}=\frac{p_{\min}T-E_{0}}{p_{\min}-p_{e}}$$

Since τ≥π(K) and $\tau \geq \tau_{l}$, with given $K\geq K_{0}$, the optimal solution to Equation (30) is obtained as follows:

(34) $$\tau^{*}(K_{0})=\max\left(\pi (K),\;\tau_{M},\tau_{l}|K=K_{0}\right)$$

The second sub-optimization problem is how to find an optimal *K* for maximizing *P_Acc_* with the initialization $\tau =\tau_{0}=\tau^{*}\left(K_{0}\right)$. From Equation (28), this sub-optimization problem is given as follows:

(35) $$\begin{array}{l} \underset{K}{\max\;}P_{Acc}\left(K\right)=\frac{T^{\prime \prime }-K\varepsilon }{T}\left(P(H_{0})\left(1-Q\left(Q^{-1}(\alpha )\xi \left({\left\{\gamma_{i}\right\}}_{i=1}^{K}\right)\text{+}\phi \left({\left\{\gamma_{i}\right\}}_{i=1}^{K}\right)\sqrt{\tau_{0}}\right)\right)+\varphi \right)\\ \text{s.t. }\psi (\tau_{0})\leq K\leq \min(\frac{T^{\prime \prime }}{\varepsilon },N),\;K\in Z \end{array}$$

where $T^{\prime \prime }=T-\tau_{0}$. From Equation (29), ψ(τ_0_) is given as follows:

(36) $$\psi (\tau_{0})=\frac{p_{c}\varepsilon -[\upsilon \left(\theta (1-\alpha )-\alpha \right)-p_{e}]\tau_{0}-\alpha \upsilon \left(\theta +1\right)T}{\upsilon \left(\theta (1-\alpha )-\alpha \right)K}$$

Since *K* is an integer, it is not computationally expensive to search through *K* from ψ(τ_0_) to $\text{min}\left(\frac{T^{\prime \prime }}{\varepsilon },N\right)$. The optimal solution to Equation (35) is calculated as follows:

(37) $$K^{*}(\tau_{0})=argmax\;\left(\;P_{Acc}\left(K\right)|\left\lfloor \psi (\tau_{0})\right\rfloor \leq K\leq \left\lceil \min(\frac{T^{\prime \prime }}{\varepsilon },N)\right\rceil \right)$$

Based on ADO and half searching, we obtain the jointly optimal solution to Equation (25) using the Algorithm 1 through alternately optimizing Equations (30) and (35). Hence, the maximum spectrum access probability is given by $P_{Acc}\left(\tau^{*},K^{*}\right)$.

**Algorithm 1** Joint optimization of τ and *K*(1)initialize *n* = 0, *K*^(*n*)^ = *K*_0_ where *K*_0_ is any integer within interval $\left(0,\;\min(\frac{T}{\varepsilon },\;N)\right)$, τ^(*n*)^ = 0 and the estimation error δ;(2)with given *K*^(*n*)^, select the *K*^(*n*)^ largest SNRs as ${\left\{\gamma_{i}\right\}}_{i=1}^{K^{\left(n\right)}}$ and obtain $\tau^{*}(K^{\left(n\right)})$ through the following steps:a)initialize $\tau_{\min}=0$ and $\tau_{\max}=T-\;K^{\left(n\right)}\varepsilon$;b)set $\tau =(\tau_{\min}+\tau_{\max})/2$;c)if $\nabla P_{Acc}(\tau )\text{==}\nabla P_{Acc}(\tau_{\min})$: let $\tau =\tau_{\min}$;d)else if $\nabla P_{Acc}(\tau )\text{==}\nabla P_{Acc}(\tau_{\max})$: let $\tau =\tau_{\max}$;e)repeat Steps (b) to (d) until $\left|\tau_{\max}-\tau_{\min}\right|<\delta$;f)set $\tau_{M}=(\tau_{\min}+\tau_{\max})/2$;g)calculate $\tau^{*}(K^{\left(n\right)})=\max\left(\pi (K),\;\tau_{M},\tau_{l}|K=K^{\left(n\right)}\right)$;(3)set τ^(*n+*1)^ = $\tau^{*}(K^{\left(n\right)})$;(4)with given τ^(*n+*1)^, obtain $K^{*}(\tau^{\left(n+1\right)})$ from Equation (37);(5)set *K*^(*n+*1)^ = $K^{*}(\tau^{\left(n+1\right)})$ and *n* = *n* + 1;(6)repeat Steps(2)~(5) until $\left|\tau^{\left(n\right)}-\tau^{\left(n-1\right)}\right|\leq \delta$ and $\left|K^{\left(n\right)}-K^{\left(n-1\right)}\right|\leq \delta$;(7)output $\tau^{*}=\tau^{\left(n\right)}$ and $K^{*}=K^{\left(n\right)}$.

## 4. Clustering Algorithm

Through the joint resource optimization, we have obtained the optimal clustering number *K* and then we will give the clustering algorithm to gather the CNs with similar locations into the same cluster. The cluster heads are elected by the fusion center in a centralized way while the common CNs join into their corresponding clusters in a distributed way. In order to select appropriate cluster heads, the fusion center must collect some information from each CN such as the distances from the fusion center and PN. Based on the distance information, the fusion center assigns the cluster head for each cluster through the clustering algorithm and then broadcasts the clustering information to all the CNs. The message broadcast by the fusion center includes the ID of the elected cluster head, the sensing time and the cooperative time.

Here, we adopt K-center clustering method to divide *D* CNs into *K* clusters and choose *K* reference nodes as the initial cluster heads. The selection of the reference nodes must focus on improving the sensing performance (*i.e.*, the distance from the PN is short) and decreasing the cooperative overhead (*i.e.*, the distance from the fusion center is short). The reference nodes are seen as *K* initial clusters and the other CNs are gathered into the nearest cluster around them. The clustering algorithm is described in Algorithm 2, where we define that $\vec{s}$ is the coordinate of node *s*, the coordinate of the cluster center is $\vec{c}=\sum_{l=1}^{L}{\vec{s}}_{l}/L$ and the distance between nodes $s_{i}$ and $s_{j}$ is $\left|{\vec{s}}_{i}-{\vec{s}}_{j}\right|$. The fusion center broadcasts the clustering information to the *K* cluster heads, then each cluster head broadcasts the beacon to its cluster nodes, and each common CN joins the corresponding cluster according to its received beacon.

**Algorithm2** Clustering algorithm(1)initialization:(a)calculate the distances between the PN$s_{PN}$ and the CN*i*$s_{i}$ as $d_{0}^{i}=\left|{\vec{s}}_{i}-{\vec{s}}_{PN}\right|$ and the distances between the CN*i* and the fusion center $s_{FC}$ as $d_{1}^{i}=\left|{\vec{s}}_{i}-{\vec{s}}_{FC}\right|$ for *i* = 1, 2, …, *D*;(b)choose 2*K* CNs with the shortest $d_{0}^{i}$ and select *K* reference nodes with the shortest $d_{1}^{i}$ from the 2*K* CNs as the initial cluster heads $v_{k}$ for *k* = 1, 2, …, *K*;(c)set the initial clusters $C_{k}=\{v_{k}\}$ and the initial cluster centers $c_{k}=v_{k}$ for *k* = 1, 2, …, *K*.(2)repeat:(a)allocate CN*i* for *i* = 1, 2, …, *D − K* to the nearest cluster *k_i_* where $k_{i}=\underset{1\leq k\leq K}{\arg\min}\left|{\vec{s}}_{i}-{\vec{c}}_{k}\right|$ and let $C_{k_{i}}=C_{k_{i}}\cup \{s_{i}\}$;(b)to each cluster, update ${\vec{c}}_{k}$ by averaging all the ${\vec{s}}_{i}$ of $s_{i}\in C_{k}$;(c)reselect the cluster head of each cluster by $v_{k}=\underset{s_{i}\in C_{k}}{\arg\min}\left|{\vec{s}}_{i}-{\vec{c}}_{k}\right|$;(d)reinitialize $C_{k}=\{v_{k}\}$ and $c_{k}=v_{k}$ for *k* = 1, 2, .., *K*;until: all the CNs in each cluster are not changed.(3)Output: $C_{k}$ for *k* = 1, 2, .., *K*.

## 5. Simulations and Discussion

In the simulations, *D* = 240 CNs are randomly placed in a 100 m × 100 m square with the fusion center located in the middle, the power of PN is $p_{s}$ = 10 mW, the noise variance is $\sigma_{n}^{2}$ = 0.1 mW, the absence and presence probabilities of PN is $P(H_{0})=P(H_{1})=0.5$, the average cooperative time overhead of each CN is ε = 0.2 ms, the sensing power is $p_{e}$ = 1.2 mW, the cooperative power is $p_{c}$ = 1 mW, the frame length is *T* = 10 ms, the battery capacity is $E_{T}$ = 15 μJ, the initial energy is $E_{0}$ = 10 μJ, the electromagnetism-to-electricity conversion efficiency is μ=0.5 and the electricity-to-electromagnetism conversion efficiency is η = 0.5.

### 5.1. Detection Performance Comparison

**Figure 6:**
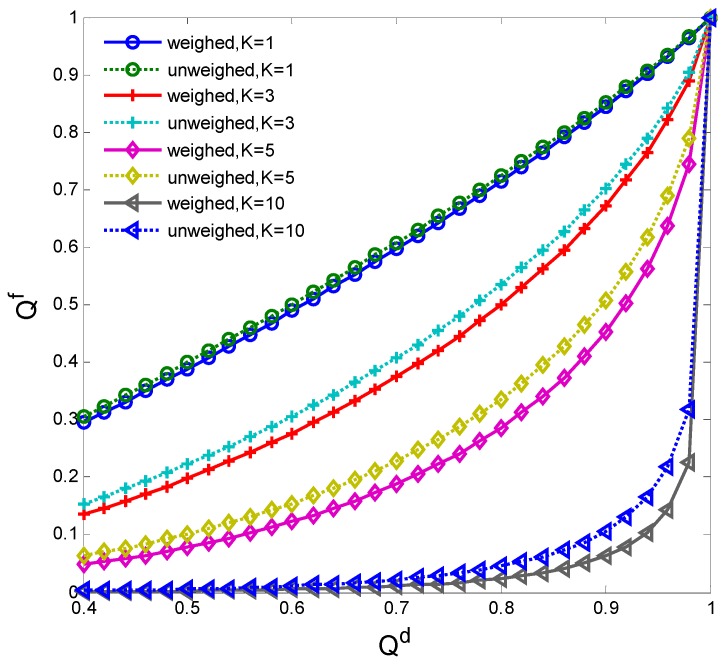
False alarm probability comparison.

Figure 6 compares the false alarm probability $Q^{f}$ of weighed and unweighed cooperative spectrum sensing with different detection probability $Q^{d}$ and the clustering number K = 1, 3, 5, 10. It is seen that $Q^{f}$ of weighed cooperative spectrum sensing is lower than that of unweighed cooperative spectrum sensing, because the local sensing performance of each CN is different and the weighed cooperative spectrum sensing may improve the combined weight of the accurate sensing information while decreasing that of the false sensing information, based on the sensing SNR. $Q^{f}$ decreases as *K* increases, which indicates that the cooperative sensing performance improves as the cooperative nodes increase. It is seen that $Q^{f}$ increases as $Q^{d}$ improves, yielding to decrease the spectrum access of the CN. Thus to improve the spectrum access, $Q^{d}$ must acquire its lower bound. It is also seen that when *K* = 1, $Q^{f}$ of weighed sensing approaches that of unweighed sensing, because if the clustering number *K* = 1, there is only one cluster head to perform spectrum sensing and thus the weighed combination has no any function on improving the sensing performance.

**Figure 7:**
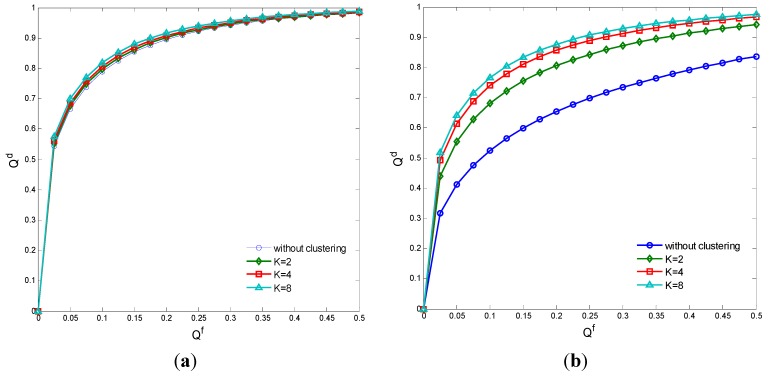
Detection probability with different clustering number. (**a**) Perfect channel; (**b**) Rayleigh fading channel.

**Figure 8:**
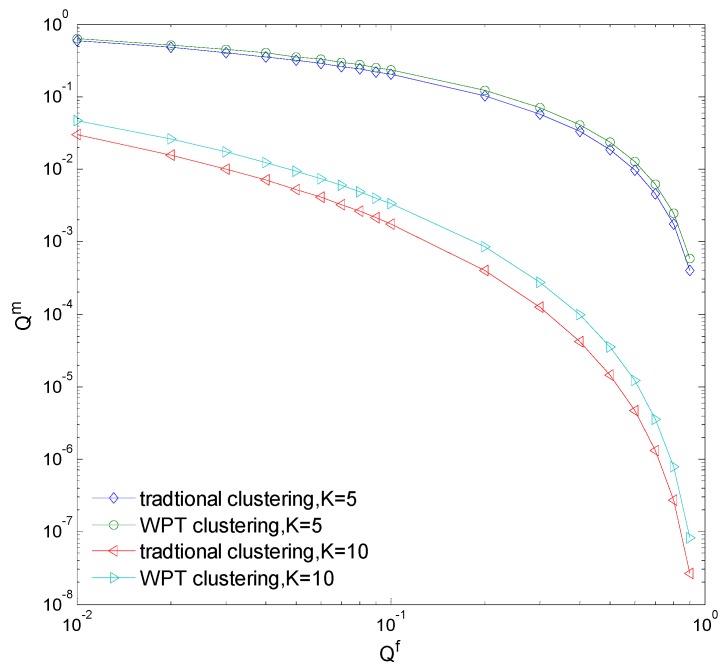
Missed detection probability.

Figure 7a,b compare the detection probability with different clustering number in a perfect channel and a Rayleigh fading channel, respectively. It is seen that the detection probability of clustering cooperative sensing is nearly same as that of the cooperative sensing without clustering in a perfect channel, because the spectrum sensing performance of each cluster head is good enough and increasing the clustering number will not improve the sensing performance too much. However, the detection probability of clustering cooperative sensing can obviously improve as the clustering number increases in a Rayleigh fading channel. Hence, we should note that the clustering cooperative spectrum sensing can only improve the sensing performance if the CN is in fading mode. Figure 8 compares the missed detection probability $Q^{m}$ of the traditional and WPT-based clustering cooperative spectrum sensing. It is seen that $Q^{m}$ of the WPT-based clustering sensing is a little larger, because the common CNs transfer wireless power instead of sensing information to the cluster head in the WPT-based clustering sensing and the combined information at the fusion center is less comprehensive. However, in the WPT-based clustering sensing, we just select the nodes with better sensing performance as the cluster heads and thus the cooperative sensing performance may approach that of the traditional clustering sensing.

**Figure 9:**
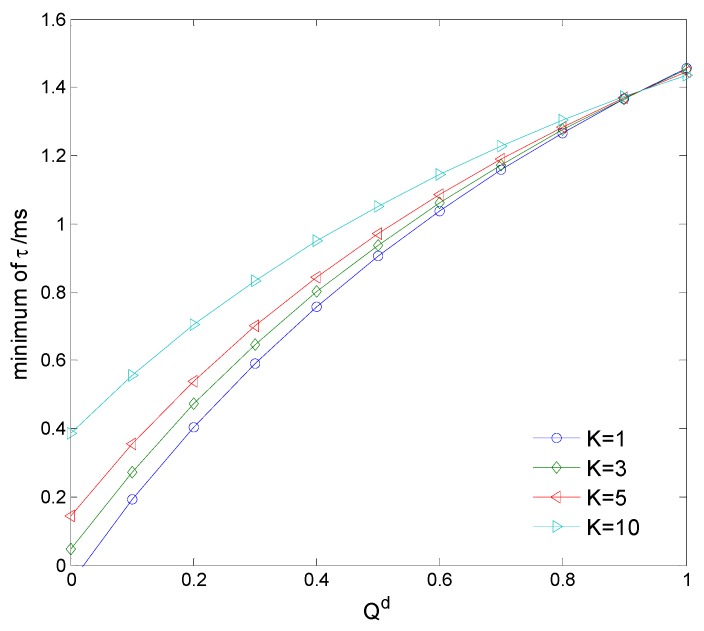
Minimum sensing time *versus* clustering number.

**Figure 10:**
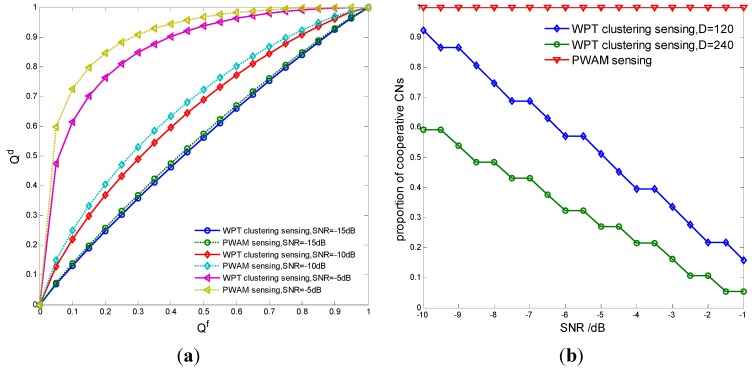
Performance comparison of different sensing methods. (**a**) Detection probability; (**b**) Proportion of cooperative CNs.

Figure 9 shows the minimum sensing time *versus* clustering number *K* = 1, 3, 5, 10. The transferred energy with the minimum τ must be enough to supply the energy used for cooperative spectrum sensing. It is seen that the minimum τ reduces with the decreasing of *K*, because the cluster nodes may increase and transfer more wireless power to the cluster head. Figure 10a,b compare the detection probability and the proportion of cooperative CNs of different spectrum sensing methods: the proposed WPT-based weighed clustering cooperative spectrum sensing and the cooperative spectrum sensing with penalty-based weight adjustment mechanism (PWAM) [27], respectively. It is seen that the detection probability of the proposed WPT-based clustering cooperative sensing is a little lower than that of the PWAM-based cooperative sensing, but the proportion of cooperative CNs in the proposed sensing model may decrease greatly as SNR increases, because we only select a few favorable cluster heads to perform cooperative spectrum sensing.

### 5.2. Transmission Performance Comparison

**Figure 11:**
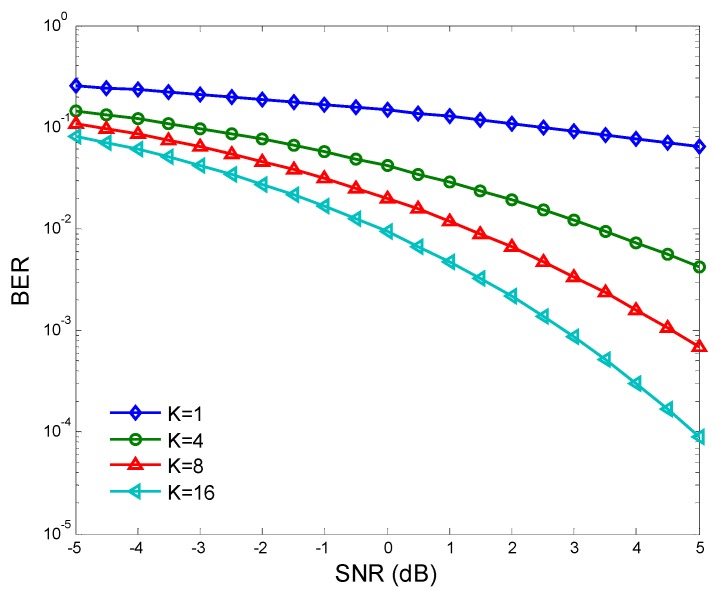
BER of reporting information.

**Figure 12:**
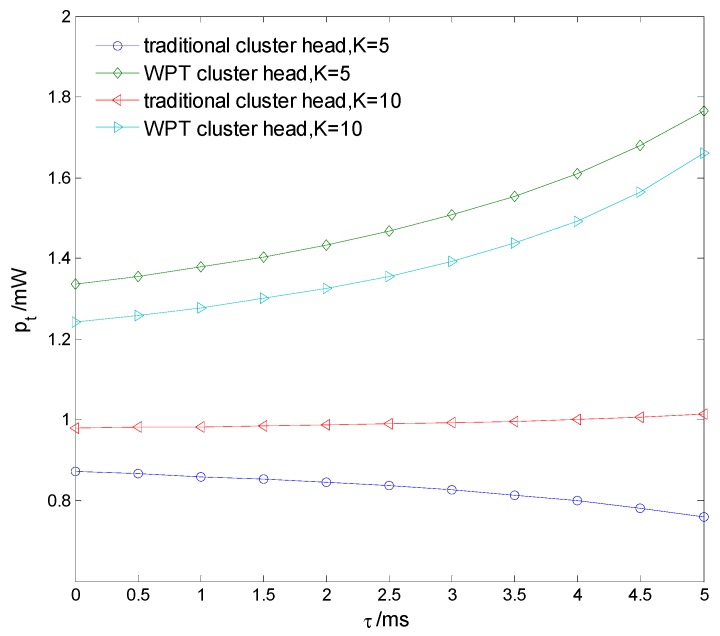
Transmission power of cluster heads.

Figure 11 compares BER of the reporting information with different clustering number. It is seen that BER decreases as the clustering number increases, because more favorable cluster heads are chosen to report sensing information to the fusion center. Figure 12 compares the information transmission power $p_{t}$ of the cluster heads in the traditional clustering cooperative spectrum sensing [12] and the proposed WPT-based clustering cooperative spectrum sensing, with different sensing time τ. It is seen that $p_{t}$ of WPT-based cooperative spectrum sensing is larger. $p_{t}$ of the traditional clustering sensing decreases as τ increases, because the dissipative sensing power increases with τ. However, $p_{t}$ of the WPT-based clustering sensing improves with τ, because the transferred PN energy also increases with τ.

**Figure 13:**
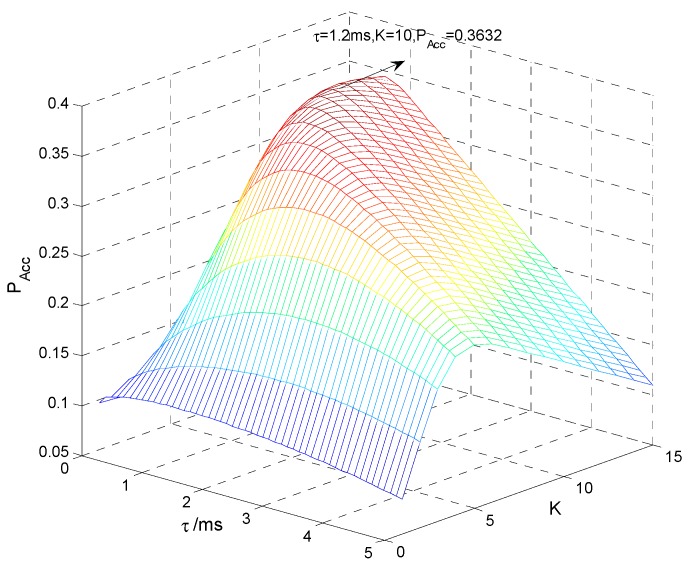
Spectrum access probability.

**Figure 14:**
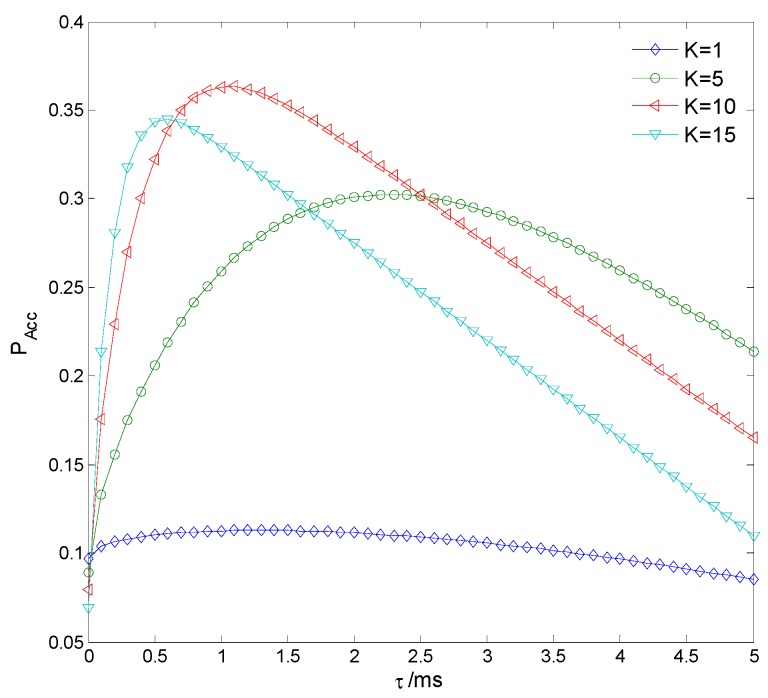
Spectrum access probability *versus* clustering number.

**Figure 15:**
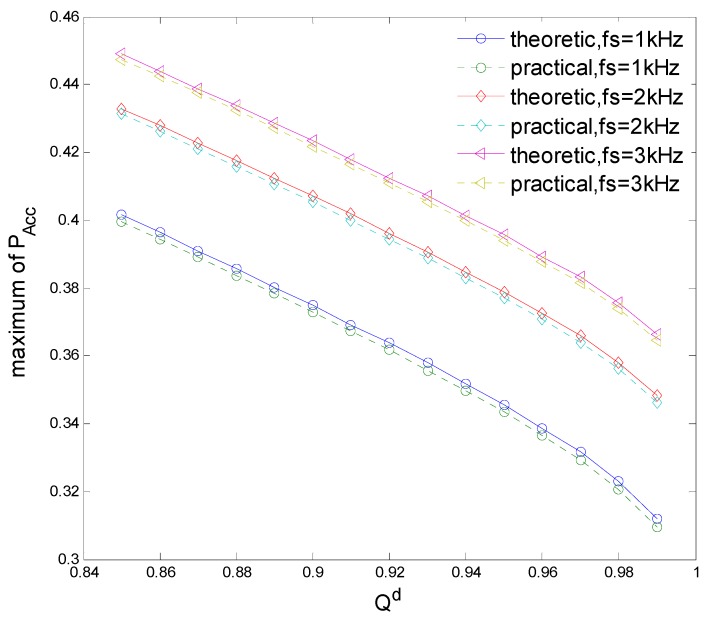
Theoretical and practical maximum spectrum access probabilities.

Figure 13 indicates the spectrum access probability $P_{Acc}$ with different sensing time τ and clustering number *K*. It is seen that there indeed exists an optimal set of τ and *K* that maximizes $P_{Acc}$, and when τ = 1.2 ms and *K* = 10, the maximum $P_{Acc}$ = 0.3632. Figure 14 shows $P_{Acc}$ *versus* *K* = 1, 5, 10, 15 with different τ, where the convex curve of $P_{Acc}$ proves the convex optimization of Equation (30). $P_{Acc}$ firstly increases and then decreases as τ increases, because the sensing performance improves while the transmission time decreases and therefore there is a tradeoff between sensing and transmission. The maximum $P_{Acc}$ of *K* = 15 is larger than that of *K* = 1 but smaller than that of *K* = 10, because the sensing performance improves while the cooperative overhead increases as *K* increases and therefore there is also a tradeoff between sensing and cooperation. Figure 15 compares the theoretical and practical maximum $P_{Acc}$ *versus* the sampling frequency *f_s_* = 1, 2, 3 kHz, with different detection probability *Q^d^*. The practical maximum $P_{Acc}$ is obtained by the proposed joint optimization algorithm. It is seen that the practical maximum accords with the theoretical maximum. $P_{Acc}$ decreases as *Q^d^* increases, because *Q^f^* increases as *Q^f^*, which decreases the spectrum access of the CN.

**Figure 16:**
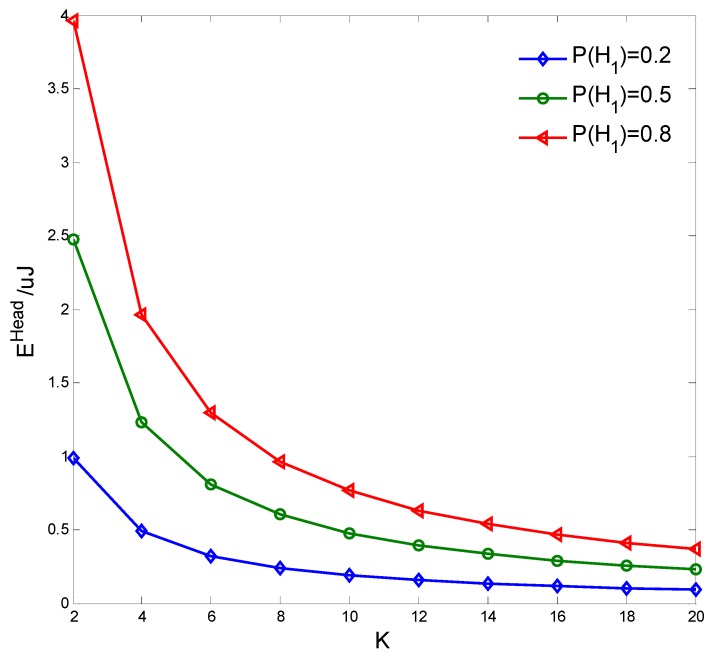
Transferred energy of cluster head.

Figure 16 shows the transferred energy of the cluster head $E^{Head}$ *versus* the presence probability of the PN $P(H_{1})$ = {0.2, 0.5, 0.8} with different clustering number *K*. It is seen that $E^{Head}$ improves as $P(H_{1})$ increases but decreases as *K* increases, because more RF energy of the PN signal will be transferred if the PN is present for a longer time, however less common CNs will transfer energy to the cluster head if the number of the cluster nodes decreases.

## 6. Conclusions

In this paper, a WPT-based weighed clustering cooperative spectrum sensing model is proposed to improve sensing performance while decreasing both cooperative overhead and energy consumption. In order to supply the electrical power for sensing and cooperation of the cluster head, the RF energy of the PN signal is converted by the common CNs of each cluster and transferred to the corresponding cluster heads. Through jointly optimizing sensing time and clustering number, the spectrum access probability of the CSN is maximized. The simulation results have shown that compared to the traditional model, the cluster head of the proposed model can achieve more transmission power and there indeed exists an optimal set of sensing times and clustering numbers that maximizes the spectrum access probability.
